# Cardiac Auscultatory Skills of Academic Family Physicians: Strength of Association with an Academic Pediatric Cardiologist

**DOI:** 10.1155/2010/370731

**Published:** 2010-11-07

**Authors:** Guzel Discigil, Ayvaz Aydogdu, Ayfer Gemalmaz, F. Serdar Gurel, Okay Basak

**Affiliations:** ^1^Department of Family Medicine, Faculty of Medicine, Adnan Menderes University, 09100 Aydin, Turkey; ^2^Department of Paediatric Cardiology, Faculty of Medicine, Adnan Menderes University, 09100 Aydin, Turkey; ^3^Ministry of Health, 06434 Ankara, Turkey

## Abstract

*Aim*. Heart murmur is common in children, and it is one of the main reasons for referral among children in primary care. The aim of this study is to evaluate agreement and consistency of normal, innocent, and pathologic murmur decision between academic family physicians and academic pediatric cardiologist. *Methods*. Seven hundred fifteen primary school children were examined by family physicians and paediatric cardiologist. Auscultatory examination was performed. Intensity, frequency, duration, quality, location, and radiation of the murmur were described if present. Agreement of normal, innocent, and pathologic murmur classification decision between family physician and paediatric cardiologist was analyzed by using kappa statistic. *Results*. Normal, innocent and pathologic murmurs were reported for 419, 228, and 54 children in family physicians' reports, respectively. Paediatric cardiologist agreed on 383 (91.4%) children as normal, 191 (83.7%) children having innocent murmur, and 19 (35.2%) children having pathologic murmur among family physician's reports. There was good consistency between family physicians and paediatric cardiologist (*κ* value = 0.679, 95% CI 0.630–0.727, *P* < .001). They agreed on the majority of normal and innocent murmur decisions. However family physicians reported pathologic murmur more frequently. *Conclusion*. Cardiac auscultatory skills of academic family physicians may be concordant with paediatric cardiologist.

## 1. Introduction

Heart murmur is a common phenomenon in as high as 50% of children, and it is one of the most common reasons for referral [1]. The role of the primary care provider is to differentiate the common innocent murmur from pathological murmur resulting from structural heart disease. Referral to the pediatric cardiologist is the next step if the nature of the murmur is unclear to the primary care physician [2]. The best way to classify a murmur as “innocent” or “pathological” is by clinical evaluation especially by careful auscultation [3]. The most helpful parameter for distinguishing innocent from pathologic murmurs is the quality of the sound. Still's murmur is the most common innocent murmur and has a typical vibratory and musical quality, whereas most pathological murmurs have much harsher grafting sound [2].

Decision process for referring as well as incidence of murmurs has not changed overtime [4]. Confidence of the examining doctor, clinical impression, anxiety level of parents and child's availability for followup are the influencing factors in decision process of referral to pediatric cardiologist [3]. Auscultatory skills of various disciplines had been evaluated. Academic general pediatricians were found to be able to correctly diagnose pathological murmurs with a similar sensitivity as compared to pediatric cardiologists. However, overall accuracy of diagnosing innocent murmurs among pediatricians was reported to be lower than pediatric cardiologists [2].

Differentiation of normal, innocent, and pathological murmurs by primary care physicians might ameliorate many parents and children anxiety and medical expenses. There are few literatures evaluating interdisciplinary consistency for cardiac auscultatory skills are some of them; conducted on trainees [5–7].

Academic family physicians participate in the training programs of the family physicians, and they play an important role in the training process. Within this respect, their own skills reflect in the training process. The aim of this study is to evaluate degree of agreement, and concordance of normal, innocent, and pathologic murmur decision between academic family physicians and pediatric cardiologist.

## 2. Methods

The study was approved by local ethical committee, and written informed consent was obtained from children's parents.

### 2.1. Study Population and Questionnaire

Seven primary schools were randomly selected among 22 primary schools in Aydin, Turkey. One in six students in each classroom was randomly chosen to participate in the study. A total of 724 students were recruited. Informed parental consent approval was obtained from 715 of them. In the final step, 715 students were evaluated first by one of the family physicians and then by the pediatric cardiologist. 

Demographic information of children such as age, grade, and residence was obtained from school records and from children themselves.

Four academic family physicians and one pediatric cardiologist were present in Aydin province. Three of the four academic family physicians and the pediatric cardiologist agreed to participate in the study. All three academic family physicians and the pediatric cardiologist were actively participating in the training program of family practice and pediatrics. Each child was examined by one of the three family physicians and the pediatric cardiologist.

### 2.2. Physical Examination and Measurements

Family physicians and pediatric cardiologist were scheduled for physical examination on different time periods. 

First, family physicians examined children. Family physicians filled out a report form including vital signs and characteristics of heart sounds. 

On the scheduled time, pediatric cardiologist blind to family physicians' reports examined the same children and filled out a separate sheet of the same report form and scheduled children with pathologic murmur for echocardiographic examination. Vital signs including height, weight, heart rate, and blood pressure were obtained. Then auscultatory examination was performed. First and second heart sounds were evaluated for intensity and appropriate splitting. Rhythm abnormalities and valvular clicks were noted if present. Periods of systole and diastole were assessed. If present, intensity, frequency, duration, quality, location, and radiation of the murmur were described. The intensity of murmurs was categorized into 6 grades of increasing loudness. Innocent murmurs were classified as Still's, venous hum, supraclavicular systemic bruit, or unclassified, and pathologic murmurs were defined. Report form sheets of the family physicians and pediatric cardiologist for each child were compared. 

Weight and height were measured following standard procedure. BMI (Body mass index) was calculated by using weight (kg)/height (m)^2^ formula [8]. Weight and height percentiles were obtained from charts of growth curves for Turkish children [9].

Arterial blood pressure was obtained by using mercury sphygmomanometer. After 10 minutes of rest in a quiet room, 3 seated blood pressure and heart rate measurements were taken at 15-minute intervals, and an average of three measurements was used in subsequent analysis.

### 2.3. Statistical Analysis

Descriptive statistics are presented as percentages, means, and standard deviations. Agreement of normal, innocent, and pathologic murmur classification decision between family physicians and pediatric cardiologist were analyzed by using kappa statistic. A *P*  value < .05 was used to indicate statistical significance. SPSS 14.0 statistical package was used for statistical analyses.

## 3. Results

A total of randomly chosen 715 children were included in the study. There were 355 (49.7%) girls and 360 (50.3%) boys in the study group. Demographic characteristics of children are shown on Table 1. 

Normal, innocent, and pathologic murmurs were reported for 419, 228, and 54 children in family physicians' reports, respectively. Pediatric cardiologist agreed on 383 (91.4%) children as normal, 191 (83.7%) children having innocent murmur, and 19 (35.2%) children having pathologic murmur among family physician's reports. Family physicians could not decide on 14 auscultatory findings where pediatric cardiologist reported 11 of them as innocent murmur, 1 pathologic murmur, and 2 of them as normal. Pediatric cardiologist could not decide on one of the cases which a family physician was reported as pathologic murmur. Table 2 shows details about decisions of family physicians and pediatric cardiologists. When concordance of decisions between family physician and pediatric cardiologist was analyzed by kappa analysis, strength of association between them was statistically significant (**κ** value = 0.679, 95% CI 0.630–0.727, *P* < .001). The results show that they agreed on the majority of normal and innocent murmur decisions. However family physicians reported pathologic murmur more frequently. Cardiac pediatrician reported 5 of the innocent murmurs as potential pathological murmur. When echocardiography was performed to these children, there was no pathological finding in any of them.

Twenty-six children (25 with pathologic murmur decision and 1 with no decision) were scheduled for echocardiography. Five children with pathologic murmur were lost at followup. Finally echocardiography was done to 21 children with pathologic murmur decision and 1 child with no decision. Fourteen (67%) of 21 children with pathological murmur decision were diagnosed as structural heart disease, and there was no pathological finding in 7 of them. Five of these children were reported as innocent murmur by family physicians. There was also no pathological finding in one child with no decision.

## 4. Discussion

We have evaluated strength of association of normal, innocent, and pathologic murmur decision between family physicians and pediatric cardiologist. Our results show that family physicians and pediatric cardiologist agreed on the majority of normal heart sounds and innocent murmurs. However, family physicians reported more pathologic murmurs which can be explained as an overcautious approach toward potential serious outcomes in the presence of cardiac pathology. 

Clinical skills of primary care physicians in the evaluation of cardiac murmurs may have vital importance and recognition of congenital heart disease in children is a diagnostic challenge. Training programs in pediatrics and family medicine should emphasize on discriminating the pathologic from the innocent heart murmur based on the history and physical examination. Even with good training and long experience, it is recognized that there will be situations in which the primary care physician will be uncertain about the presence or absence of heart disease [10]. There is no published study comparing auscultatory skills of primary care physicians and pediatric cardiologists to our knowledge.

Clinical examination by a pediatric cardiologist is a sensitive and specific way to discriminate innocent murmur from congenital heart disease [11]. Further investigations have focused on the question of whether generalist can do as well [12–14]. Diagnosis before referral to pediatric cardiologist has been reported to reach only near to 20% among general practitioners [7]. In two studies reported by Rajakumar et al. and Haney et al., general pediatricians were about as sensitive to the heart disease as the pediatric cardiologists, but the specificity of the generalists was poorer [12, 13]. Furthermore the diagnostic accuracy of heart murmurs by pediatricians especially their specificity for the diagnosis of heart disease is suboptimal [11]. On the other hand, family practice trainees had low identification rate for cardiac events [5].

Primary care physician serves a gatekeeper role in the evaluation and referral of children with heart murmur. Many parents may not be aware of that and the majority of childhood heart murmurs can be innocent and transient. Parental anxiety is common among parents of children with heart murmur [15]. Distinguishing innocent murmur from pathological murmur in primary care would help to improve parental anxiety in general. Furthermore, better training of family physicians in cardiac auscultation may help in reducing medical expenses [16]. Skills in clinical assessment of heart murmurs in children can be improved among general practitioners in training [7]. The result of our study shows that there may be good concordance between academic family physicians and pediatric cardiologist auscultatory findings. Even though there is no published literature proving the effect of diagnostic accuracy for cardiac problems in children among family physicians, we believe that identifying the majority of heart murmurs by family physicians would help to improve health outcomes in general especially in areas with high children population and in rural areas where it may not be easy to reach pediatric cardiologist. Results of our study show that family physicians may identify majority of normal children and children with an innocent murmur, and more cautious decision is an expected approach in the presence of pathologic murmur. 

There are some limitations of our study. The conclusion drawn from the data collected by a single group of family physicians and pediatric cardiologist can be safely generalized to others with similar training and auscultatory skills. However, the data of this study might not reflect the diagnostic accuracy of family physicians and pediatric cardiologists in general. Further research is needed on diagnostic accuracy, sensitivity, and specificity of cardiac auscultatory skills of family physicians practicing in the field and pediatric cardiologists or general pediatricians. Despite its limitation in this scope, this study has important implications for child health in primary care.

## 5. Conclusion

Conclusions of this study reflect the situation in which family physicians and pediatric cardiologist have done a preliminary evaluation and concluded that the murmur warrants echocardiography. It is a common approach that echocardiography can be done only to a minority of children since prevalence of heart murmur is very high and the majority of them are innocent murmurs. Therefore better training of primary care physicians in cardiac auscultation may be of great benefit for primary health care. Our results reveal that cardiac auscultatory skills of family physicians may be strongly associated with pediatric cardiologists. However higher percentage of undecided and pathologic murmur reports among family physicians shows us that there is still need for improvement of the family physicians auscultatory skills.

## Figures and Tables

**Table 1:** Demographic characteristics of 715 children.

Features	Demographics
Age (mean ± SD)	10.4 ± 2.5 years
Girls (*n* %)	355 (49.7%)
Boys (*n* %)	360 (50.3%)
Height percentile (mean ± SD)	48.1 ± 28.5%
Weight percentile (mean ± SD)	42.6 ± 27.7%
BMI (mean ± SD)	17.4 ± 3.1 kg/m²
Pulse rate (mean ± SD)	94.3 ± 14.4/minute
Systolic blood pressure (mean ± SD)	112.5 ± 11.8 mmHg

Diastolic blood pressure (mean ± SD)	73.2 ± 7.4 mmHg

**Table 2:** Auscultatory findings of family physicians and pediatric cardiologist.

	Pediatric cardiologist					
		Normal	Innocent murmur	Pathologic murmur	Cannot decide	Total
Family physicians	Normal	383 (53.6%)	36 (5.0%)	0	0	419 (58.6%)
	Innocent murmur	32 (4.5%)	191 (26.7%)	5 (0.7%)	0	228 (31.9%)
	Pathologic murmur	8 (1.1%)	26 (3.6%)	19 (2.7%)	1 (0.1%)	54 (7.6%)
	Cannot decide	2 (0.3%)	11 (1.5%)	1 (0.1%)	0	14 (2.0%)

Total	425 (59.4%)	264 (36.9%)	25 (3.5%)	1 (0.1%)	715 (100%)
